# Biopanning of polypeptides binding to bovine ephemeral fever virus G_1_ protein from phage display peptide library

**DOI:** 10.1186/s12917-017-1315-x

**Published:** 2018-01-04

**Authors:** Peili Hou, Guimin Zhao, Chengqiang He, Hongmei Wang, Hongbin He

**Affiliations:** Key Laboratory of Animal Resistant Biology of Shandong, Ruminant Disease Research Center, College of Life Sciences, Shandong Normal University, No. 88 East Wenhua Road, Jinan City, Shandong Province China

**Keywords:** Bovine ephemeral fever virus, Phage display random peptide library, G_1_ binding peptide, Antiviral peptide

## Abstract

**Background:**

The bovine ephemeral fever virus (BEFV) glycoprotein neutralization site 1 (also referred as G_1_ protein), is a critical protein responsible for virus infectivity and eliciting immune-protection, however, binding peptides of BEFV G_1_ protein are still unclear. Thus, the aim of the present study was to screen specific polypeptides, which bind BEFV G_1_ protein with high-affinity and inhibit BEFV replication.

**Methods:**

The purified BEFV G_1_ was coated and then reacted with the M13-based Ph.D.-7 phage random display library. The peptides for target binding were automated sequenced after four rounds of enrichment biopanning. The amino acid sequences of polypeptide displayed on positive clones were deduced and the affinity of positive polypeptides with BEFV G_1_ was assayed by ELISA. Then the roles of specific G_1_-binding peptides in the context of BEFV infection were analyzed.

**Results:**

The results showed that 27 specific peptide ligands displaying 11 different amino acid sequences were obtained, and the T18 and T25 clone had a higher affinity to G_1_ protein than the other clones. Then their antiviral roles of two phage clones (T25 and T18) showed that both phage polypeptide T25 and T18 exerted inhibition on BEFV replication compared to control group. Moreover, synthetic peptide based on T18 (HSIRYDF) and T25 (YSLRSDY) alone or combined use on BEFV replication showed that the synthetic peptides could effectively inhibit the formation of cytopathic plaque and significantly inhibit BEFV RNA replication in a dose-dependent manner.

**Conclusion:**

Two antiviral peptide ligands binding to bovine ephemeral fever virus G_1_ protein from phage display peptide library were identified, which may provide a potential research tool for diagnostic reagents and novel antiviral agents.

**Electronic supplementary material:**

The online version of this article (10.1186/s12917-017-1315-x) contains supplementary material, which is available to authorized users.

## Background

Bovine ephemeral fever (BEF) caused by bovine ephemeral fever virus (BEFV), also known as 3 day fever, is an acute febrile infection of cattle and water buffaloes. The typical clinical symptoms of BEF are characterized by the rapid onset and rapid recovery of clinical signs, such as fever, depression, muscle stiffness, oral and nasal discharges, joint pain and lamenes [1–3].The disease is more frequently distributed in a vast expanse of the world including tropical, subtropical and temperate regions of Africa, the Middle East, Australia and Asia, and leads to serious economic losses to the cattle industry through loss of milk production, significant impacts on trade [4–6]. To date, no specific treatment and drug is available for BEFV.

BEFV belongs to the genus *Ephemerovirus* in the family *Rhabdoviridae*, and consists of a single stranded, negative-sense RNA genome with a lipid envelope and five structural proteins [7, 8]. The G protein plays an important role in inducing neutralizing antibodies, and it is also closely associated with cell tropism, pathogenicity, fusion and interaction with host cellular receptors [5, 9–12]. The protein G consists of five major neutralisation sites (G_1_,G_2_,G_3a_,G_3b_,G_4_) defined by BEFV G protein specific monoclonal antibodies (MAbs) in competition ELISAs, and among these sites, site G_1_ composed of two minimal B cell epitopes is a linear neutralisation site involving in eliciting neutralizing antibodies. It is reported that site G_1_ is predicted to face the viral membrane in both the pre-fusion and post-fusion forms of the trimer and may be approachable to antibodies [13–15]. Therefore, G_1_ protein can be used as a potential target for antiviral drug screening.

Phage display, a well-established powerful and popular technology, has been extensively used in many fields, including antibody engineering [16, 17], ligand screening [18, 19], peptide drug discovery and manufacture [20, 21], disease molecular diagnostic analysis [22], biosensing [23] and vaccine research and development [24]. The high capacity and abundance of random phage display library makes it appropriate for high-throughput screening of peptide ligands that specifically bind with the given targets. In virology, several peptides have been identified as potential antivirals against classical swine fever virus [25], transmissible gastroenteritis virus [26], influenza virus [27], newcastle disease virus [28], human immunodeficiency virus (HIV) [29] and hepatitis B virus [30] using phage display technology. However, there is no report about screening of polypeptides binding to BEFV by phage display library.

In this study, we identified specific peptides that were capable of binding to the G_1_ protein of BEFV from the 7-mer phage display library using the immobilized BEFV G_1_ protein as a target, and we analyzed their roles in the context of BEFV infection in vitro, which is of great significance for potential diagnosis and treatment of BEF.

## Methods

### Virus, cells and plasmid

Ph.D.-7 phage-displayed peptide library was purchased from New England Biolabs (Ipswich, MA, USA). The titer of this peptide library was up to 2 × 10^13^ plaque forming units (pfu/mL), containing about 2.8 × 10^9^ independent random peptide sequences. BEFV(Shandong/China/2011) were isolated and stored by the Ruminant Disease Research Center, Shandong Normal University, Jinan, Shandong Province, China, and the 50% tissue culture infected dose (TCID_50_) of BEFV determined by Reed-Muench method were 5.0 × 10^5.0^TCID_50_/mL. Anti-BEFV antibody extracted from cattle was preserved in our laboratory. More concretely, clinical serum samples were collected from detected dairy cattle cases of BEFV infections in China. The antibodies against BEFV were identified by the microneutralization assay according to the instructions of the Agricultural Industry Criteria of the People’s Republic of China (publication no. NY/T543–2002). The anti-BEFV antibodies in this study were applied to analyze the bioactivity of purified recombinant G_1_ protein in Western Blot. BHK-21 cells were incubated in Dulbecco’s modified Eagle’s medium (DMEM, Invitrogen, Carlsbad, CA, USA), supplemented with 10% fetal bovine serum (FBS, Gibco, USA) at 37 °C, 5%CO_2_. Prokaryotic expression vector pET-32a(+) plasmid were maintained in our laboratory. The G genes of Shandong/China/2011 (GenBank: JX234571.1) was previously described [31], and the pMD18-T-G was used as template for PCR amplifying the G_1_ gene using sense primer G1-F:5’-CGGGATCCAGAGCTTGGTGTGAATAC-3′ and antisense primer G1-R:5’-CCGCTCGAGCCAACCTACAACAGCAGATA-3′. Underlined parts of primers reprensent *Bam* HI and *Xho* I restriction sites, respectively. The amplified G_1_ gene region and the points linked to the primers used were shown in Supplement Figure one with the size of 420 bp. The PCR product was cloned into the pET-32a(+) vector and the plasmids with G_1_ gene was sequenced by BGI (BGI, China).

### Expression and purification of BEFV recombinant G_1_ protein in *E. coli*

The recombinant plasmid pET-32a(+)-G_1_ was transformed into high efficiency chemical competent cells *E.coli* BL21(DE3) pLysS (TransGen Biotech Company, Beijing, China). BL21 cells were cultured in LB medium containing 100 mg/L Ampicillin. The LB medium was shaken at 37°C until the OD_600_ reached 0.6–0.8, then isopropyl-d-thiogalactoside (IPTG) was added to a final concentration of 1 mmol/L to induce G_1_ expression. The BL21 cells were further incubated for 6 h at 37 °C, and harvested by centrifugation at 8000×g for 5 min at 4 °C.The precipitation of BL21 cells were resuspended in lysis buffer including 10 mmol/L phosphate buffered saline (PBS, pH 7.4), imidazole (10 mmol/L), lysozyme (10 g/L), trypsin inhibitor (10 g/L), pepsin inhibitor (1.0 g/L) and PMSF (1.0 g/L) for sonication on ice. The cell lysate was centrifuged at 12,000×g for 20 min at 4 °C. The supernatant was collected and poured in a 10 mL Ni^2+^-Sepharose 6 Fast Flow column pre-equilibrated with lysis buffer. The column was then washed with imidazole (10, 50 and 100 mmol/L) in PBS (10 mmol/L, pH 7.4) to remove nonspecifically bound proteins, and eluted with 250 mmol/L imidazole. Meanwhile, the designated pET32a(+) vector control protein was also expressed and purified in *E. coli* in the same condition. The yield protein concentration was measured using the Pierce BCA-200 Protein Assay Kit. To verify the reactivity of purified G_1_ protein, we conducted western blotting using purified G_1_ protein and pET32a(+) vector control protein as antigen by incubating with anti-BEFV positive antibody (diluted at 1:1000) for 2 h, and then with goat anti-cattle IgG conjugated to peroxidase (diluted at 1:2000, Abcam,ab102150,USA). Full immunoblots was visualized by enhanced chemiluminescence immunoassay (ECL kit, Thermo Fisher Scientific Inc., USA).

### Biopanning and enrichment analysis of phage library using BEFV G_1_ as target

The Ph.D.-7 peptide library (NEB, New England Biolabs, USA, E8100S) was used to screen for G_1_ binding peptides according to the manufacturer’s instructions with slight modifications. In short, 96-well plates were coated with 100 μL/well of the recombinant G_1_ protein at a concentration of 100 μg/mL, biopanning was carried out by incubating the phage display library (10^11^ phages/mL). After washing away the unbound phage with Tris-buffered saline (TBS + 0.1% Tween 20), a bound phage was eluted in TBS and titrated as described in the standard protocol, and then subjected to the next round of panning. The second, third and fourth rounds of panning were done under more stringent conditions using less amount of the target (100 μL/well of a solution at 75,50,25 μg/mL of the recombinant G_1_ protein, and shorter incubation time (1.5 h, 1 h and 0.5 h in the 2, 3 and 4th round, respectively), and washed with higher concentrations of Tween 20-TBS (0.25%, 0.5% and 0.5%, respectively) for longer time (10 × 2 min, 10 × 3 min and 10 × 4 min). Inbetween each round of panning, the titer of the panning phages in binding buffer (here referred to as input) and that in the elution buffer (here referred to as output) was determined, and the ratio of output to input was analyzed to assess the enrichment efficiency. After four rounds of panning, individual clones were randomly picked and amplified in *E. coli* ER2738 strain to prepare their DNA.

### Sequencing of positive phage clones

Individual positive phage clones were amplified, and phage single-stranded DNA was extracted and purified using M13 Phage DNA Rapid Extraction Kit (Spin-column, Signalway Biotechnology, USA), DNA was sequenced by Sangon sequencing (Sangon Biotech Co.,Ltd., Shanghai, China) using the-96 gIII sequencing primer 5′-CCCTCATAGTTAGCGTAACG-3′ provided by the Ph.D.-7 peptide library kit. Amino acid sequences were deduced from phage display peptide DNA sequences by ExPASy Translate tool (http://web.expasy.org/translate/). The polypeptides used in the study were synthesis and purified by the Beijing Science and Technology Co., Ltd. and an irrelevant control peptide (AEMLELS) acquired from another screening strategy was synthesized by China Peptides Co., Ltd. (Shanghai, China).

### Binding analysis of individual phage using ELISA

The phage displayed peptide clones with different amino acid sequences were subjected to ELISA. Briefly, ELISA plates were coated with the G_1_ protein at a concentration of 10 μg/well diluted in 0.1 mol/L NaHCO_3_ (pH 8.6) overnight at 4°C. Meanwhile, the purified pET32a(+) vector control protein coated with the same concentration was used as control group. All ELISA plates were blocked with 200 μL blocking buffer (5% BSA) for 2 h at room temperature. Ten-fold serial dilutions of the selected phages were added to both coated plates starting with 10^12^ phages in the first well. Plates were incubated for 2 h at room temperature and then washed 6 times with TBST (TBS containing 0.5% Tween-20). Bound phage was then detected with 200 μL 1:1000 diluted horseradish peroxidase (HRP)-conjugated anti-M13 antibody (GE Healthcare, 27–9421-01, USA) in blocking buffer. Finally, the peroxidase activity was rapidly detected with substrate solution TMB upon addition of a sulfuric acid stop solution. The absorbance of the reaction was determined at 450 nm with an ELISA microplate reader (Shanghai Utrao Medical Instrument Co., Ltd., Shanghai, China).

### Antiviral activity of positive phage clones and synthetic peptides

BHK-21 cells (1 × 10^5^/well) were plated in 12-well culture plates to 90% confluence. Amplified positive phage clones at concentrations of 10^10^(pfu/mL) and synthetic peptides at concentrations of 0, 10, 20, 40, 80, 160, and 320 μg/mL were incubated with 100TCID_50_/0.1 mL BEFV at 37°C for 1 h, respectively, and then a mixture of virus and amplified positive phages or various concentrations of synthetic peptides transferred onto the cell monolayers for adsorption for 2 h. The medium was then replaced with maintenance medium containing 2%FBS in 1 mL DMEM and incubated for a further 36 h. The treated cells and their supernatants were collected followed by three cycles of alternate freezing and thawing and stored at −80 °C before use.

### Plaque reduction assay

Vero cells (1 × 10^5^ cells/well) were seeded into 12-well plates for 12 h maintained in Dulbecco’s modified Eagle’s medium (DMEM, Invitrogen, USA), supplemented with 10% inactivated fetal bovine serum (FBS, Gibco, USA) and a mix of the antibiotics Penicillin (100 IU) and Streptomycin (100 μg/mL) at 37°C, 5%CO_2_. Synthetic peptides at concentrations of 0, 10, 20, 40, 80, 160, and 320 μg/mL were incubated with 100TCID_50_/0.1 mL BEFV at 37°C for 1 h, and then transferred onto the cell monolayers. After 2 h of incubation, discarding the supernatant, the cells were incubated under normal culture conditions at 37°C for 36 h. The monolayers were spread out with 2% low melting agarose, and formation of cytopathic plaque was visualized by staining the cell monolayers with 1%(*W*/*V*) neutral red solution (Sigma-Aldrich, USA). The effects of peptides on BEFV infection were evaluated by counting the plaques and calculating the titer (pfu/mL) and inhibitory rate.

### Real-time reverse transcription polymerase chain reaction (RT-PCR)

Total RNA was extracted from 200 μL treated samples using QIAamp® Viral RNA Mini Kit (QIAGEN, Valencia, CA, USA) and eluted into final volumes of 50 μL. Reverse transcription was performed using PrimeScript RT reagent Kit (TaKaRa, Japan) according to manufacturer’s protocols. The SYBR Green-based quantitative real-time RT-PCR was carried out using a Premix Ex Taq kit according to the manufacturer’s protocols (TaKaRa, Japan) in a total volume of 25 μL on a Roche LightCycler 480 Real Time PCR System (Roche Applied Science, Germany). The sequences of primer sets used in real-time RT-PCR with specific primers for BEFV N gene and endogenous glyceraldehyde 3-phosphate dehydrogenase (GAPDH) gene were listed in Table 1. Real-time RT-PCR was carried out as follows: 95 °C for 5 min, followed by 40 cycles of 94 °C for 20 s, 60 °C for 30 s and 30 s at 72°C. The threshold cycle (Ct) value related to RNA levels of each target gene was normalized to GAPDH expression and the relative expression of each sample was analysed by the 2(^−△△Ct^) method.

**Table 1:** The sequences of primers used in real-time RT-PCR

Gene		Sequences (5′-3′)	Amplicon size(bp)
BEFV N	N-F	TCCTTCACCATGTACTGCAC	138
	N-R	ACCTTGTGGCACTCTCAAC	
GAPDH	GAPDH-F	TCATGACCACAGTCCATGCC	144
	GAPDH-R	GGATGACCTTGCCCACAGCC	

### Statistical analysis

Each treated sample was run in triplicate. Statistical analysis was performed by one-way analysis of variance and values were considered significant when *p* < 0.05. Figures were performed using the GraphPad™ Prism 5.0 software (Graphpad Software Inc., San Diego, CA, USA).

## Results

### Purification of recombinant BEFV G_1_ protein in *E. coli*

**Fig. 1:**
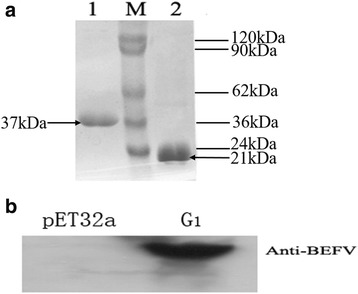
SDS-PAGE and Western blot analysis of purified recombinant G_1_ protein. Large amounts of highly expressed recombinant G_1_ protein were purified using Ni^2+^-sepharose 6 Fast Flow affinity media under native conditions. **a** SDS-PAGE analyses of purified recombinant G_1_ protein and pET32a vector protein. M, molecular protein marker; Lane 1, purified recombinant G_1_ protein; Lane 2, pET32a vector protein. **b** Western blot analysis of purified recombinant G_1_ protein and pET32a vector protein react with BEFV antibody. The left lane: vector protein control; the right lane: purified recombinant G_1_ protein

The G_1_ gene region of 420 bp (Additional file 1: Figure S1) was amplified by PCR and cloned into pET32a(+) vector. G_1_ protein was expressed in *E.coli* strain BL21 (DE3) and it mainly existed in the supernatant of the bacterial lysate, which indicated that G_1_ gene had been efficiently expressed in prokaryotic expression system in a soluble form. Large amounts of highly expressed protein were purified using Ni^2+^-sepharose 6 Fast Flow affinity media. The results of SDS-PAGE indicated that we obtained the highly-pure recombinant G_1_ protein with an expected size of ~ 37 kDa. Meanwhile, the expected thioredoxin protein encoded by empty pET-32a (+) vector alone was also clearly visible with the size of ~ 21 kDa (Fig. 1a). Western blot were performed to analyze the bioactivity of purified recombinant G_1_ protein. The result indicated that purification of recombinant G_1_ protein could immunoreact with anti-BEFV antibody (Fig. 1b). Therefore, those data were indicated that the purified G_1_ used in this study was biologically active. The concentration of purified G_1_ and control pET-32a (+) vector protein determined by the absorbance at 280 nm were 1.65 mg/mL and 2.85 mg/mL.

### Identification of phage-displayed peptides with BEFV G_1_ protein

In this study, a Ph.D.-7™ Phage Display Peptide Library was used to screen high-affinity ligands that could selectively bind to G_1_ protein. Following four rounds of biopanning, the amounts of input phages and output phages were determined by titration, and the properties of phage recovery efficiency (output phages/input phages) in each round were calculated. As shown in Table 2, the recovery efficiency was 195-fold higher (from 7.2 × 10^−3^ to 3.7 × 10^−5^) after four rounds of panning than after the first round, suggesting that the phages specifically bound to G_1_ protein were successfully enriched.

**Table 2:** Enrichment of phage peptides from library by BEFV G_1_ protein biopanning

Item	First round biopanning	Second round biopanning	Third round biopanning	Fourth round biopanning
G1(μg)	10	7.5	5	2.5
Concentration of Tween20 (*v*/v)	0.1%	0.25%	0.5%	0.5%
Input/PFU (pfu/ml)	1 × 10^11^	1 × 10^11^	1 × 10^11^	1 × 10^11^
Output/PFU (pfu/ml)	3.7 × 10^6^	5.2 × 10^7^	8.7 × 10^7^	7.2 × 10^8^
Recovery (Output/input)	3.7 × 10^−5^	5.2 × 10^−4^	8.7 × 10^−4^	7.2 × 10^−3^

### DNA sequencing of the selected phage clones

After the 4th panning round, a total of 27 phage clones were randomly selected and amplified. The DNA of selected positive phage clones was extracted and sequenced. The DNA sequencing reports showed that 11 deduced peptides were finally obtained among the 27 phages and presented in Table 3. Alignment of the peptides revealed probably putative G_1_ binding conservative consensus motifs. Phages 4, 11, 12, 23 and 25 had a consensus sequence YSLRSDY named T25, which bear YSLR similarity with other 11 phage clones. The overlapping motif sequence derived from Phages 3 and 18 was HSIRYDF named T18.

**Table 3:** Sequencing results of positive phage colonies with specifically binding to BEFV G_1_ protein

Groups	Phage clones	Phage displayed peptide DNA sequence	Phage displayed peptide amino acid sequence	Poly-peptide names
1	4, 11, 12, 23, 25	TATTCTCTTCGTTCTGATTAT	YSLRSDY	T25
2	5, 6, 20	TATTCTCTTCGTCAGGATTGG	YSLRQDW	T20
3	1, 13, 19	TATAGTTTGCGTACTGATTGG	YSLRTDW	T19
4	16, 22, 26	TATTCTCTTCGTCAGGAGAGG	YSLRQER	T26
5	14, 24	TATAGTTTGCGTGCGGATCGT	YSLRADR	T24
6	10, 17	GCTCTGAGTAGTCTGCGTAAT	ALSSLRN	T17
7	3, 18	CATTCTATTCGGTATGATTTT	HSIRYDF	T18
8	2, 15	CATAGTATTCGTGTTGATTGG	HSIRVDW	T15
9	21	GATTGGATTTTTCCTGCGTTT	DWIFPAF	T21
10	9, 27	AAGGTTTGGATTGTTCCTTCT	KVWIVPS	T27
11	7, 8	AAGGTTTGGTTGCTTCATTCT	KVWLLHS	T8

### Binding analysis of individual phage with G_1_ using ELISA

**Fig. 2:**
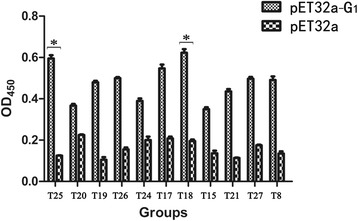
ELISA analysis of binding activities between the selected phages and BEFV G_1_ protein. 11 selected phages labeled T25,T20,T19,T26,T24,T17,T18,T15,T21,T27 and T8 were incubated with recombinant protein G_1_ and pET32a vector control protein in ELISA plates to determine individual binding activities as measured by OD_450_ values. The OD_450_ readings were performed in triplicates. Statistical significance (*p* < 0.05) is noted by “*” compared to control groups

In order to test the binding ability of the selected phage clones, 11 independent phage clone groups encoding different amino acid sequences were selected and detected by ELISA using the purified recombinant G_1_ protein as target. As shown in Fig. 2, the absorbance obtained from each phage combined with the target G_1_ protein was significantly higher than that of the control pET-32a (+) vector protein. Among these G_1_ binding peptides, T18 and T25 showed the higher binding ability to G_1_ protein when compared to the other clones. Although there is no criterion for judging the positive clones, it is suggested that the T18 and T25 clone might have a higher affinity to G_1_ protein than the other clones.

### Inhibition of peptides on BEFV replication

**Fig. 3:**
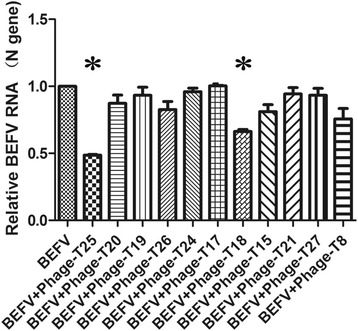
Inhibitory effects of selected phage on BEFV detected by real-time RT-PCR. 11 selected phages labeled Phage T25,T20,T19,T26,T24,T17,T18,T15,T21,T27 and T8 were incubated with 100TCID_50_/0.1 mL, respectively. The SYBR Green-based quantitative real-time PCR was used to determine BEFV RNA with specific primers for N gene. The relative expression of BEFV was normalized to GAPDH expression and analysed by the 2(^−△△Ct^) method. Each treated sample was run in triplicate. Statistical analysis was performed by one-way analysis of variance and values were considered significant when *p* < 0.05. Figures were performed using the GraphPad™ Prism 5.0 software. Statistical significance (*p* < 0.05) was noted by “*” compared to control groups

To further determine whether the phages that displayed G_1_-binding peptides have the potential to inhibit BEFV infection, we performed virus-blocking assays in BHK-21 cells. Briefly, 100 TCID_50_/0.1 mL BEFV was incubated with individual clone with a titer of 10^9^pfu/0.1 mL that displayed G_1_-binding peptide or irrelevant phage, respectively, and then inoculated into BHK-21 cells. Meanwhile, the non-phage treated BEFV was included as a control. After incubation for 36 h, the SYBR Green-based real-time RT-PCR was performed to determine the RNA copies of BEFV in different phage-treated cells. The results showed that T25 and T18 peptides significantly exerted antiviral activity, and the phage polypeptide T25 could reduce BEFV RNA copy number by 2 times, while T18 decrease by 1.5 times compared to control group. Obviously, T25 was the greatest antiviral peptide among all these selected peptides (Fig. 3).

**Fig. 4:**
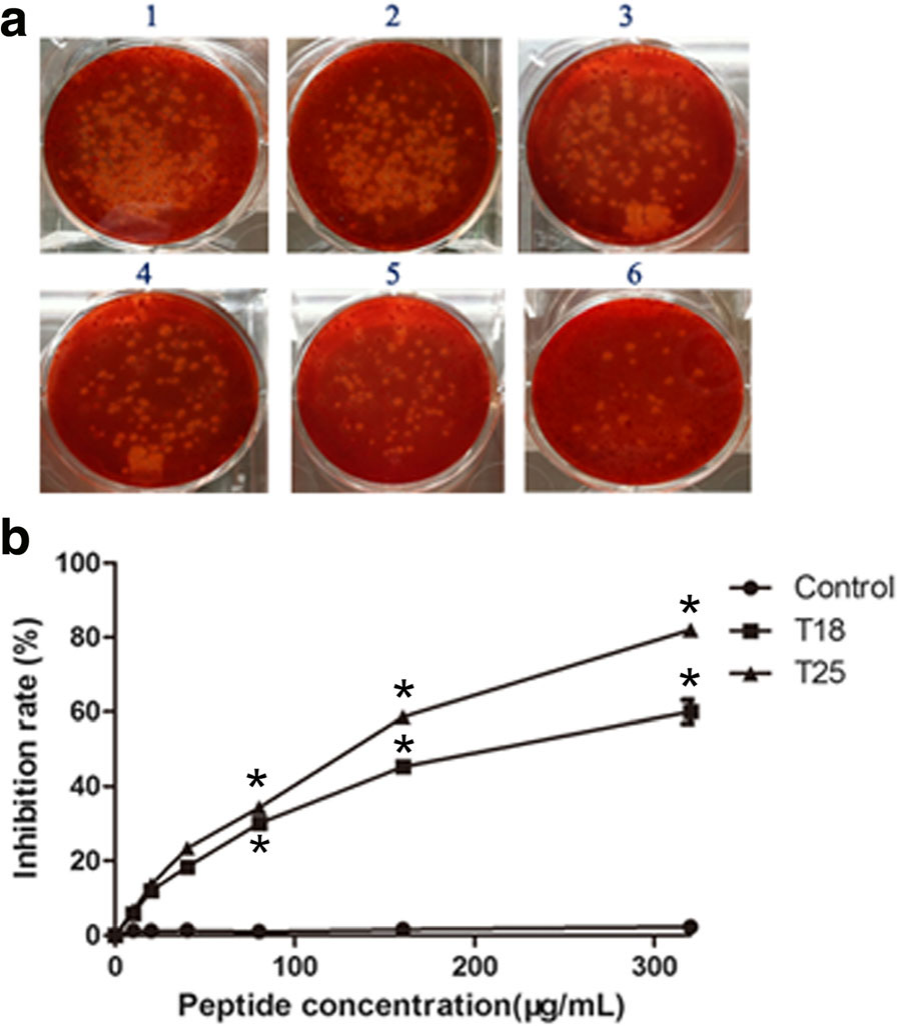
Inhibition of peptide T18 and T25 to plaque production on Vero cells. **a** Inhibition ability of synthetic peptides T25 was determined by the reduce plaque assay. 100 TCID50/0.1 mL BEFV and 2-fold serial diluted synthetic peptides ranging from 10 μg/mL to 320 μg/mL were pre-inoculated and then inoculated Vero cells.1, BEFV; 2, BEFV + 20 μg/mL;3, BEFV + 40 μg/mL; 4, BEFV + 80 μg/mL; 5, BEFV + 160 μg/mL; 6, BEFV + 320 μg/mL. **b** Synthetic peptides T18 and T25 at 2-fold serial dilution concentrations of 10-320 μg/mL were incubated with 100 TCID_50_/0.1 mL BEFV, respectively. The synthetic irrelevant peptide (AEMLELS) acted as control. Antiviral activities of the peptides were determined by calculating the percentage of inhibitory rate compared against the controls using the following formula; IN (%) = T/C × 100 [42], where, T is the mean of the number of plaque treated with different concentration of peptides. C is the mean of the number of plaque treated with corresponding synthetic irrelevant peptides that did not react with G_1_ protein. Data presented were the mean OD values (±SD) of triplicate samples. Figures were performed using the GraphPad™ Prism 5.0 software. Statistical significance (*p* < 0.05) was noted by “*” compared to control groups

To further investigate the antiviral activity of selected polypeptide (T18 and T25), firstly, we synthesized the peptides borne by phages T18 and T25 and conducted the dose-dependent effect of synthetic peptides T18 and T25 on inhibition of BEFV replication by virus plaque assay. At indicated concentration points, the reduce plaque assay showed the synthetic peptides could significantly inhibit the formation of cytopathic plaque at a concentration of 80 μg/mL, while the group of 320 μg/mL nearly abolished plaque formation in Vero cells, and the reduce plaque result of serial diluted synthetic peptides T25 was shown in Fig. 4a. Then antiviral activities of synthetic peptides T18 and T25 were determined by calculating the percentage of inhibitory rate compared against the controls. The results suggested that the similar trend was at work on the inhibition of T18 and T25 on BEFV infection. It turned out that 80 μg/mL, 160 μg/mL and 320 μg/mL of synthetic peptides could significantly interfere with BEFV formation of cytopathic plaque (*p* < 0.05) (Fig. 4b).

**Fig. 5:**
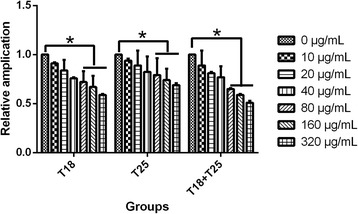
Antiviral activities of synthetic polypeptides on BEFV infection detected by real-time RT-PCR. Synthetic polypeptides T18, T25 and T18 + T25 were incubated with 100TCID_50_/0.1 mL, respectively. The SYBR Green-based quantitative real-time PCR was used to determine BEFV RNA with specific primers for N gene. The relative expression of BEFV was normalized to GAPDH expression and analysed by the 2(^−△△Ct^) method. Each treated sample was run in triplicate. Statistical analysis was performed by one-way analysis of variance and values were considered significant when *p* < 0.05. Figures were performed using the GraphPad™ Prism 5.0 software

We further analyzed dose dependence of antiviral activity of the synthetic peptide T18 and T25 alone, and compared the synergistic effects of combined use on BEFV replication. As shown in Fig. 5, the results of real time RT-PCR showed that 320 μg/mL, 160 μg/mL and 80 μg/mL of synthetic peptides alone and combined use could significantly inhibit BEFV RNA replication (*P* < 0.05), and both T18 and T25 exerted inhibition on BEFV replication of BHK-21cells in a dose-dependent manner. However, the effects of peptides T18 and T25 combined were similar to that of peptide T18 or T25 alone (*P* > 0.5) (Fig. 5).

## Discussion

The study of the molecular mechanism of virus infection is the basic and vital approach for prevention and control of the disease. As for BEFV infection, it has been proposed that the attachment of virus to the host cell membrane via the interaction of the viral envelope G protein with cell surface receptors is considered to be the initial step of BEFV infection [32]. And G_1_ protein is considered to be the main protective antigen of BEFV that only reacts with sera against BEFV, currently some molecular and serological diagnostic methods to detect BEFV were established based on G_1_ protein [12, 33]. In this study, we aimed at utilizing G_1_ protein as target protein for screening its ligands and preliminary investigating the role of binding peptides during BEFV infection. The highly effective prokaryotic system is exemplified an optimal choice for heterologous protein, and the BEFV G_1_ protein was purified in a moderately form of soluble (Fig. 1a), ensuring the target protein’s native configuration in vitro. Furthermore it can specifically react with anti-BEFV antibodies as an antigen protein (Fig. 1b). Taken together, the G_1_ glycoprotein used in this study is biologically active.

In recent years, using purified protein or whole virus particle as the targets of phage peptide library screening provides an ideal approach to identify multifunctional peptides [22, 34–36]. In this study, we used a phage-displayed heptapeptide library to identify G_1_ protein interactors, and the conditions of the selection were strictly limited to enrichment specific G_1_-binding phages(Table 2). After four rounds of panning, 11 deduced peptide amino acid sequences from 27 selected candidate phage clones exhibited high affinity to G_1_ protein according to ELISA (Fig. 2). And these peptide sequences showed partial consensus motifs (Table 3), indicating that the screening process was successful. Meanwhile, partial consensus motifs which is possibly typical functional motifs of more complex proteins would be promising to discover the ligands of G_1_ glycoprotein and analyze their roles in the context of BEFV infection. In addition, there has no report about BEFV receptor, and the consensus motif may mimic the discontinuous binding site as receptor agonists/antagonists or regulate the biological function of receptors [37–39].

Phage-display technology provides an effective way of identifying peptides with affinities to specific proteins for diagnostic analysis and the discovery of mimic antibodies [22, 40]. As the phages bearing the specific affinity peptides to BEFV G_1_ protein, they have the potential to develop a phage-mediated diagnostic assay to BEFV. It is reported that the BEFV G_1_ protein located at the C-terminal stalk of the G protein is one target of virus-neutralizing antibodies, and G_1_ protein is a linear neutralization site (Y487-K503) that comprises two minimal B cell epitopes [5, 11].The potential significant achievement of our work is the discovery of mimic antibodies, synthetic peptide in vitro could be used as a specific antibody mimic to detect BEFV in virus-infected cells.

Peptide ligands targeting a specific protein surface obtained by phage-display technology are widely used as therapeutic agents by interfering with protein-protein interactions [20, 21, 25–29, 41]. Interestingly, the phage clone T18 and T25 exhibited binding affinity to G_1_ protein and antiviral activity against BEFV (Fig. 3), whereas other phages with high affinity to G_1_ protein did not effectively inhibit the replication of BEFV. This means that the binding affinity of other phages do not necessarily connection with antiviral activity. In addition, a possible explanation of antiviral activity is that the G_1_ protein binding phage ligand mediates an inhibitory effect of BEFV infection by competitive binding to receptor binding sites or neutralizing epitopes and thus inhibits the virus attachment/entry. In order to exclude the effect of phage itself on protein binding and antiviral activity against BEFV, we synthesized T18 and T25 peptides in vitro and conducted the reduce plaque assay in vero cells that significantly formed cytopathic plaque during BEFV infection. The results showed that the synthetic peptides could significantly inhibit the formation of cytopathic plaque(Fig. 4) and inhibit BEFV replication in a dose dependent way (Fig. 5). Altogether, T18 and T25 peptides are potentially potent antiviral molecules worthy of further development and test. This study provides an inexpensive way to discover functional motif for BEFV infection using the phage-display technology, which is possible to find the potential protein to mediate the interaction between BEFV and the host.

## Conclusion

In summary, we successfully isolated G_1_ protein binding peptides from phage display library. Antiviral effect assay showed that G_1_-binding peptides were dose-dependently effective against BEFV infection at the cellular level, which might have potential applications in the diagnosis and treatment of BEFV.

## Additional file

Additional file 1: Figure S1. Schematic drawing of BEFV G and the points linked to the primers of cloned G_1_ used. (A) The number of amino acids region about full length G gene and Region G_1_ (from 390 aa–529 aa) were indicated. (B) Presentation of G_1_ nucleotides sized and gene sequence amplification locus and positions of the forward (G1-F) and reversed (G1-R) primers used in this study. (TIFF 11694 kb)

## Abbreviations

BEF: Bovine ephemeral fever
BEFV: Bovine ephemeral fever virus
BHK-21: Baby Hamster Syrian Kidney
BSA: Bovine serum albumin
DMEM: Dulbecco’s modified Eagle’s medium
ELISA: Enzyme linked immunosorbent assay
FBS: Fetal bovine serum
GAPDH: Glyceraldehyde 3-phosphate dehydrogenase
IPTG: Isopropyl-d-thiogalactoside
NEB: New England Biolabs
PBS: phosphate buffered saline
PMSF: Phenylmethanesulfonyl fluoride
RT-PCR: Reverse transcription polymerase chain reaction
TBS: Tris-buffered saline
TCID_50_: 50% tissue culture infected dose
