# Supplementary figures and images for: Biopanning of polypeptides binding to bovine ephemeral fever virus G1 protein from phage display peptide library
Source: BMC Vet Res. 2018 Jan 4;14:3. doi: 10.1186/s12917-017-1315-x (PMC5753476; doi:10.1186/s12917-017-1315-x)

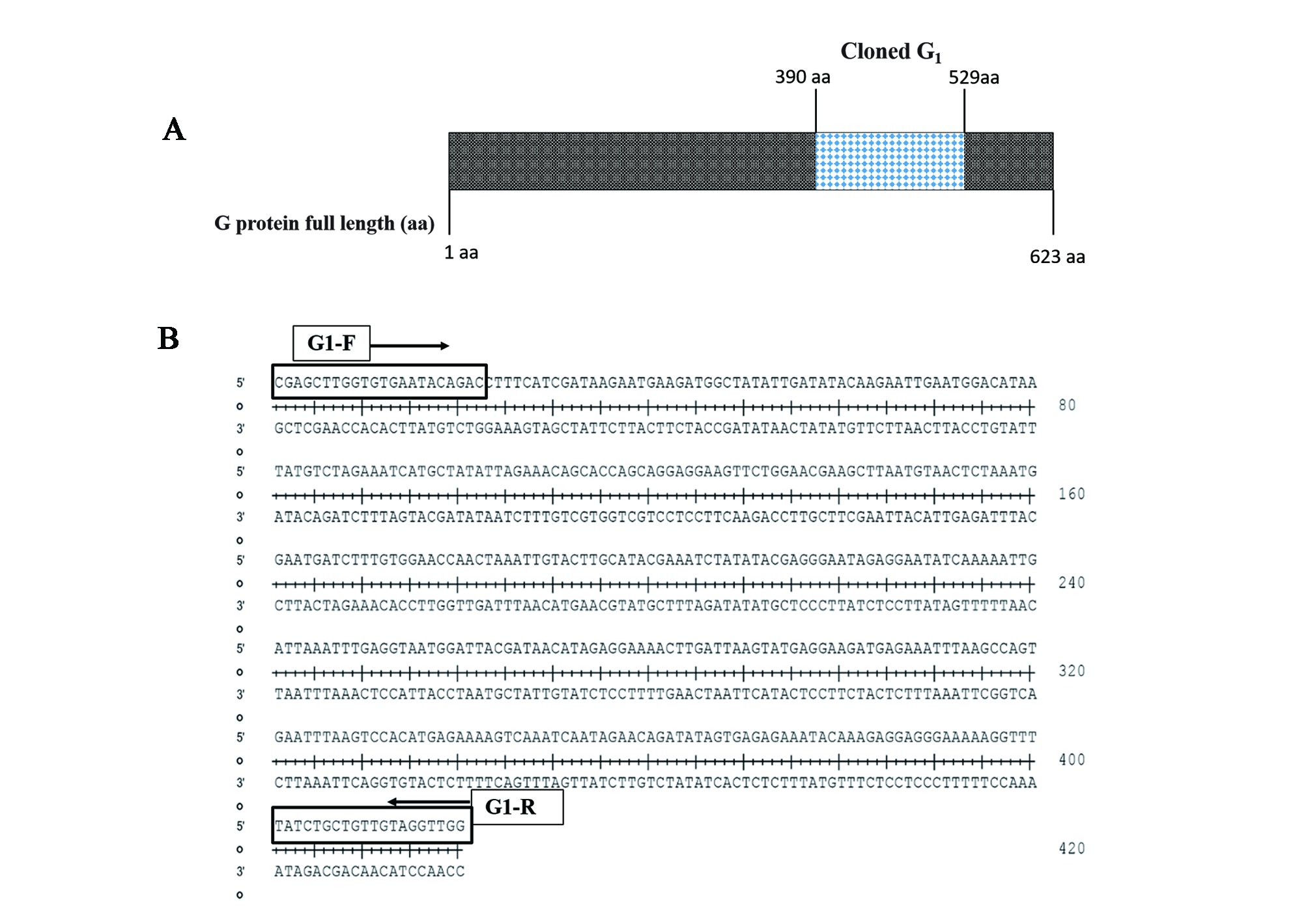

Supplement: Additional file 1: Figure S1. — Schematic drawing of BEFV G and the points linked to the primers of cloned G1 used. (A) The number of amino acids region about full length G gene and Region G1 (from 390 aa–529 aa) were indicated. (B) Presentation of G1 nucleotides sized and gene sequence amplification locus and positions of the forward (G1-F) and reversed (G1-R) primers used in this study. (TIFF 11694 kb) [file 12917_2017_1315_MOESM1_ESM.tif]
